# Multi-Robot Path Planning for High-Density Parking Environments Considering Efficiency and Fairness

**DOI:** 10.3390/s25144342

**Published:** 2025-07-11

**Authors:** Jinhyuk Lee, Woojin Chung

**Affiliations:** Department of Mechanical Engineering, Korea University, Seoul 02841, Republic of Korea; leejh9826@korea.ac.kr

**Keywords:** path planning, multi-robot, automated valet parking

## Abstract

As parking congestion at airport parking lots intensifies, high-density parking (HDP) systems with multiple parking robots are gaining attention for improving operational efficiency. However, conventional multi-agent pathfinding (MAPF) methods primarily focus on overall efficiency improvement, often neglecting the priority of individual parking tasks. Additionally, these methods assume robots are ideal agents, resulting in physically infeasible paths for parking robots. We propose a multi-robot path planning approach that balances efficiency and priority. The proposed method improves priority-based search (PBS) by dynamically adjusting priorities, thereby ensuring both operational efficiency and priority of individual vehicles. A simulator replicating a real airport parking environment with 100 parking slots and parking robots under development was implemented to validate the approach. Real-world parking data from an airport was used as input, demonstrating that the proposed autonomous parking system can effectively handle peak-season parking demand. The proposed method achieves a throughput exceeding 41 vehicles per hour with appropriate weight value, meeting the peak-season demand while maintaining acceptable fairness. Our approach provides a practical foundation for establishing time-based parking operation strategies and estimating the number of robots recommended for a given parking scenario.

## 1. Introduction

### 1.1. Research Background

The rapid increase in air travel after COVID-19 has intensified parking congestion at Incheon Airport in South Korea. In the summer 2024, the parking occupancy rate far exceeded 100%, often leaving customers without available spaces. In some cases, customers even missed their flights while searching for an empty parking spot. The shortage of parking spaces has also led to severe double parking issues, obstructing traffic flow within the parking lot. Although temporary solutions such as increased parking fees have been implemented, a more fundamental and systematic approach is urgently required.

High-Density Parking (HDP) [1] systems have been studied as a solution to parking congestion in airports and densely populated urban areas. HDP aims to improve parking density by eliminating the need for human-operated parking, adopting autonomous parking systems instead. HDP removes pedestrian pathways, including aisles and drop-off areas, redesigning the parking layout to use space more efficiently [2,3]. A similar concept can be applied to alleviate airport parking shortages with multiple parking robots.

Multi-Agent Path Finding (MAPF) [4] has emerged as a key technique for planning paths in multi-robot systems. MAPF has been extended in various ways, including planning in continuous time [5], scaling to large environments [6], and handling dynamic priorities [7]. Most methods in the literature aim to enhance the system efficiency and do not consider the priority of individual tasks. Moreover, robots are typically modeled as simplified agents, assuming idealized motion. To the best of our knowledge, no existing work in the field accounts for the dynamic constraints of parking robots.

**Figure 1:**
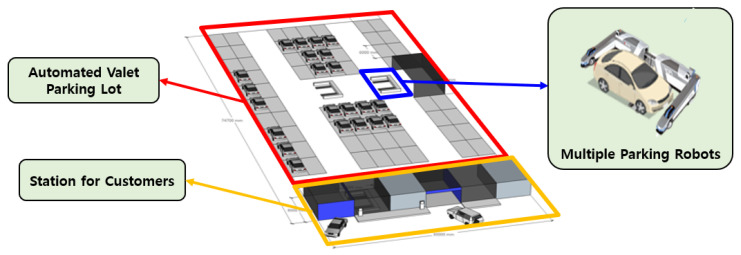
Autonomous parking system with HDP and parking robots.

As shown in Figure 1, a valet parking system using multiple parking robots can replace manual parking operations at airports. The robots approach vehicles from the side, aligning their gantries with the wheels to lift up and transport the vehicles to designated parking slots. Drivers drop off and retrieve their cars only through designated stations, and they are not allowed inside the parking area. Only parking robots are allowed to operate inside the unmanned parking space, autonomously handling vehicle placement. In such automated valet parking systems, pedestrians or human drivers are not permitted. Therefore, it can be assured that no dynamic obstacles exist within the parking area. A predefined map of the parking lot is used, and the occupancy status of each parking slot is monitored in real time. The server monitors the overall state of the parking facility via sensors installed in the environment and on the robots, and it plans paths in real time considering sensor feedback. A site implementing such an automated parking system is currently under construction at Incheon Airport.

**Figure 2:**
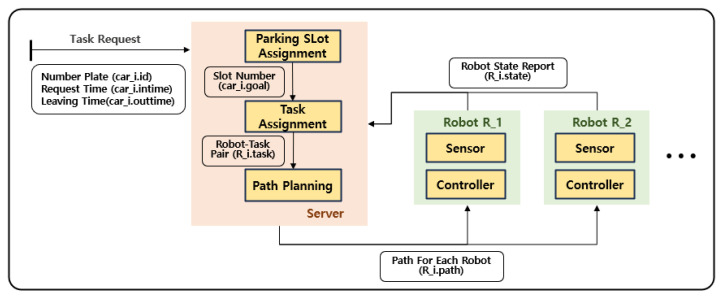
Multi-robot parking system.

In order to utilize multiple parking robots without any collisions, a path planning system for multiple robots is required. Figure 2 shows a multi-robot parking system consisting of a central server and multiple parking robots. When a customer requests a service, a task is generated, including the request time and vehicle identification number. Since the parking location is not predetermined, a parking slot allocation stage is necessary. The server assigns the vehicle an available slot to enhance the overall efficiency of the parking lot based on factors such as expected parking duration. Next, a task allocation stage assigns robots to tasks. Robots are assigned to tasks while considering factors such as customer wait time. Once the task allocation stage is completed, each robot now has its own designated task. However, if each robot plans its movement independently, collisions may occur. In order to prevent this from happening, a multi-robot path planning stage is required. An effective multi-robot path planning algorithm is essential to guarantee safe and efficient operation within the high-density parking environment.

The first two stages primarily aim to improve efficiency, and there remains room for further enhancement. However, task execution becomes infeasible without collision-free paths for multiple robots, making path planning the most critical challenge. Therefore, we mainly focus on the path planning stage among the three stages of generating multi-robot paths in this study. The other two stages are handled with basic functional modules and are briefly explained in Section 3.

The main contributions of this work are as follows. First, we extended the multi-agent path finding framework to parking scenarios, accounting for the characteristics of autonomous parking systems, as explained in Section 1.2. In addition, the method can generate paths balancing both operational efficiency and individual task priority. Finally, real robot dynamics including acceleration and rotational motion were reflected, enabling more accurate time-based path planning. The proposed method was validated through simulations based on a real-world autonomous parking lot modeled after Incheon Airport.

### 1.2. Attributes of Multiple Parking Robot Path Planning Problem

Multi-robot path planning algorithms usually assume ideal agents, disregarding the dynamic constraints of real robots. Additionally, the algorithms focus on optimizing overall efficiency rather than considering the priority of individual tasks. Therefore, these conventional multi-robot path planning algorithms are more suitable for environments like warehouses, where customers are not waiting on the spot. In contrast, we need to deal with multiple parking robot path planning problems in this study. In such problems, the following attributes from the perspectives of robots, tasks, and environment must be considered.

The present study incorporates the characteristics of the parking robots currently under development at Incheon Airport into the path planning process. In order to ensure the safety of vehicle transportation, parking robots need to operate with low acceleration. Assuming the constant velocity of robots may lead to significant temporal errors. Additionally, robots cannot perform turning maneuvers while moving but can rotate only with zero linear velocity. Parking robots have omnidirectional mobility, which allows for movement in any direction without changing orientation.

Unlike multi-robot tasks in warehouses, parking operations involve customers waiting on the spot. If a parking task requested later is processed first, customers who arrived earlier in the waiting line may express dissatisfaction. The same issue arises during vehicle retrieval, where prioritizing a later request over an earlier one can lead to customer complaints. The psychological response in queues is not limited to airport parking lots but is also observed in crowded urban environments, such as parking lots in office buildings and restaurants. Taking the aspects of parking tasks into account, a balance between priority and efficiency must be considered for parking robots. Strictly following request orders ensures fairness but may reduce operational efficiency. Conversely, prioritizing efficiency alone may lead to unfair task handling, creating a trade-off between the two factors.

**Figure 3:**
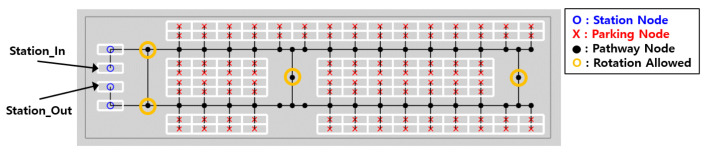
Topological map of the automated parking lot.

The automated parking lot specifications currently under development at Incheon Airport were fully incorporated into the topological map shown in Figure 3. The map consists of parking nodes, travel nodes, station nodes, and edges connecting them. The assumed environment measures 120.5 m in width and 30 m in length, accommodating 100 parking spaces with one entry and one exit station. Due to narrow corridors, robots can rotate only at designated nodes and must follow directional constraints when entering parking slots.

## 2. Related Work

### 2.1. Multi-Agent Path Finding for Multi-Robot Coordination

Multi-Agent Path Finding (MAPF) [4] has been widely studied as a core method to find paths for multiple robots. Existing MAPF approaches are largely categorized into centralized and decentralized methods, based on whether a central server is needed. Decentralized MAPF has been developed for environments where communication between robots is limited or where the number of agents is too large for centralized control. This study addresses a parking system with the central server and does not involve thousands of robots, making only centralized MAPF relevant to the scope of the research. Centralized MAPF methods can be further divided into optimal and suboptimal approaches. Optimal MAPF exhaustively searches all possible solutions to guarantee the best result. On the other hand, suboptimal MAPF sacrifices optimality to lower computational costs and allows for faster planning with acceptable performance.

Among the optimal MAPF algorithms [8], state-of-the-art methods are either variants of Conflict-Based Search (CBS) [9] or they employ strategies similar to CBS. CBS adopts a two-level search architecture. At the high level, a conflict tree is explored to generate constraints that resolve conflicts between robots. The low level searches for individual paths for each robot that satisfy the assigned constraints. Several extensions have been proposed to overcome the limitations of CBS. One notable example is Continuous Conflict-Based Search (CCBS) [5], which integrates Safe Interval Path Planning (SIPP) [10] to enable planning in continuous time domains. Another optimal MAPF approach, Increasing Cost Tree Search (ICTS) [11], also follows a two-level search scheme. In ICTS, the high level explores an increasing cost tree to identify feasible solutions. Symmetry reasoning techniques for optimal MAPF were proposed in [12], which significantly improved scalability by reducing node expansions.

A trade-off exists between computation time and solution quality in MAPF. Bounded suboptimal MAPF addresses this trade-off by allowing for slight degradation in solution quality to reduce planning time. The goal is to find a solution whose cost does not exceed w times the optimal cost, where w is referred to as the suboptimality factor. Bounded suboptimal MAPF methods are typically derived from optimal MAPF algorithms. Suboptimal ICTS [13] based on ICTS and Enhanced CBS (ECBS) [14] that integrates focal search into CBS are such examples. Further improvements have led to Explicit Estimation CBS (EECBS) [6], which enhances ECBS by incorporating the cost estimation method.

In unbounded suboptimal MAPF, some methods extend bounded approaches by setting the suboptimality factor as infinity. Other unbounded suboptimal methods include rule-based algorithms and prioritized planning. PIBT [15] is a rule-based method that guarantees a solution in polynomial time on bi-connected graphs without blockage. In prioritized planning [7,16], paths are computed sequentially according to priority order. Lower-priority robots avoid the reserved paths of higher-priority ones by treating them as obstacles. Unbounded suboptimal methods are highly scalable but often yield lower quality or incomplete solutions. To mitigate the issues, incorporating Large Neighborhood Search (LNS) [17] to MAPF has been proposed as a post-processing technique. Recent studies have explored multi-objective approaches that improve efficiency by allowing for controlled violations of priority constraints [18]. Another recent hierarchical approach combines a probabilistic roadmap with reinforcement learning to efficiently generate paths under non-holonomic constraints [19].

### 2.2. MAPF Methods Considering Priority

The starting point for MAPF research based on prioritized planning was marked by [16]. The study introduced Cooperative A (CA*), a search method with predefined priorities. In CA*, higher-priority robots plan their paths first by searching through a space–time domain composed of two space axes and a one time axis. The planned paths are stored in a three-dimensional reservation table. Lower-priority robots then treat these reserved paths as obstacles during their planning process. However, this method may fail to find a solution even when one exists due to fixed priorities. Additionally, the use of static priority assignments can lead to less efficient solutions.

Priority-Based Search (PBS) was proposed by [7] to address the limitations of [16]. PBS eliminates the need to predefine priorities among agents by employing a two-level search. In the high-level search, a priority tree is used to determine the priority order of robots. Each robot plans its path in the low-level search while avoiding the reserved paths of higher-priority robots. However, like other MAPF methods, PBS assumes robots to be ideal agents. This assumption makes it unsuitable for addressing the dynamic constraints of parking robots. Additionally, since PBS uses depth-first search and terminates as soon as a solution is found, it does not consider improvements in solution quality.

The Token Passing (TP) algorithm proposes a scalable planning method in decentralized environments [20]. Agents sequentially receive a token that grants them the right to plan their path while avoiding conflicts with previously planned paths. TP-SIPPwRT was suggested in [21] to extend TP by integrating SIPP with reservation tables. TP-SIPPwRT enables efficient collision avoidance under the continuous-time domain. However, the performance is highly dependent on the order in which agents receive the token, often leading to unfair path quality. Deadlocks might occur in constrained spaces due to the decentralized framework. The system also relies on reliable communication between agents. Delays or failures in token transmission can influence overall performance. Due to these limitations, no existing MAPF algorithm can be directly applied to the problem addressed in the current study.

### 2.3. Planning Approaches in High-Density Parking (HDP) Systems

There are several studies that specifically address the planning problem in high-density parking environments [22]. Ref. [23] proposed a method to assign parking locations based on whether a vehicle’s exit time information is available. A parking layout optimization approach for a k-stack parking system was introduced in [24]. The study aimed to minimize both vehicle relocation frequency and exit times, supported by simulation results. Various parking space allocation strategies were suggested and evaluated in [25]. The study considered factors such as arrival time, clustering, and blockage probability.

However, all of the studies mentioned above assume that every vehicle is capable of autonomous parking without the assistance of parking robots. The parking system must have full control over all vehicles, which is not realistic in practice. Moreover, most existing vehicles are not equipped with autonomous parking functionality, making them incompatible with such systems. In addition, subtle variations in dynamic constraints, such as vehicle size and wheelbase, make it highly complex to generate individual paths for each vehicle. Therefore, for the practical operation of an autonomous parking lot, the deployment of parking robots is essential. A multi-robot path planning algorithm that takes the characteristics of parking robots into account is required to support such systems.

## 3. Methodology

As discussed earlier, the initial two stages are managed by basic functional modules, while the primary focus of the study lies in the path planning stage. In the parking slot assignment stage, pickup location and delivery location of a task is decided. When a vehicle enters the parking lot, the pickup location is the station, and the delivery location is assigned randomly. When a vehicle exits the parking lot, the pickup location corresponds to the parking slot where the vehicle was initially parked, and the delivery location is the designated station. In the task assignment stage, each task is allocated to the robot with the shortest estimated completion time. The travel time between all pairs of nodes is calculated in advance. The calculated result serves as an approximation, as it does not account for potential collisions with other robots. Task assignment is performed by assigning each task to the robot according to the estimated time.

**Figure 4:**
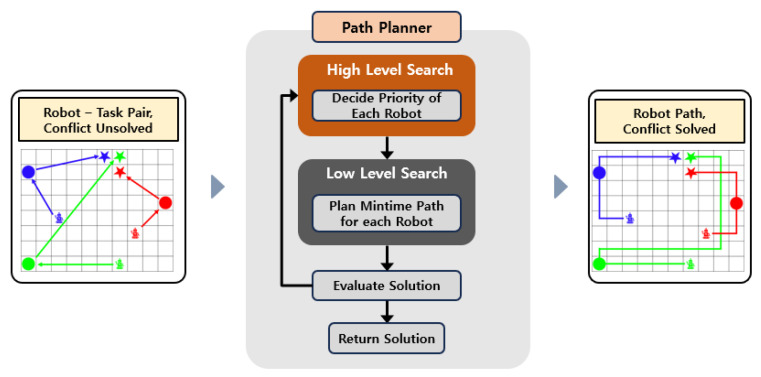
Path planning module for multiple parking robots.

In the path planning stage, the input consists of robot–task pairs produced by task allocation, which do not account for potential collisions. The module must generate collision-free paths for multiple robots based on the input. In the present study, the path planning module was designed by modifying the search strategy of PBS. The module consists of two layers—a high level and a low level—as illustrated in Figure 4.

### 3.1. High-Level Search

**Figure 5:**
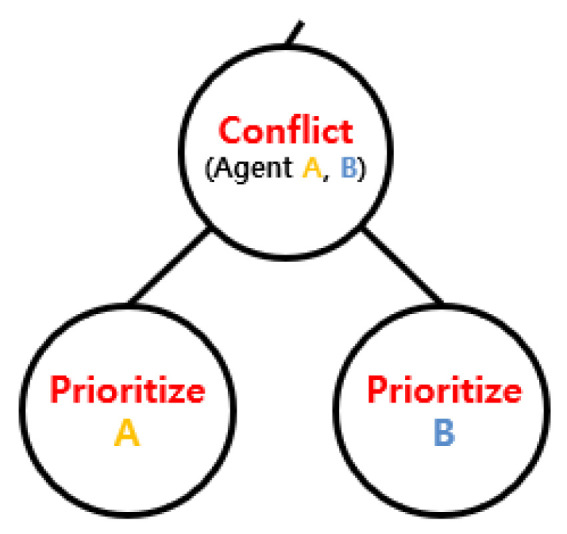
Priority tree.

The high-level search explores robot priorities using the Priority Tree (PT) shown in Figure 5. Each node in the PT contains the priority order among robots, along with the solution consisting of individual paths. For N robots, there are N! possible priority orders. The high-level search navigates the tree to determine an appropriate priority ordering that enables collision-free execution of each task.

**Figure 6:**
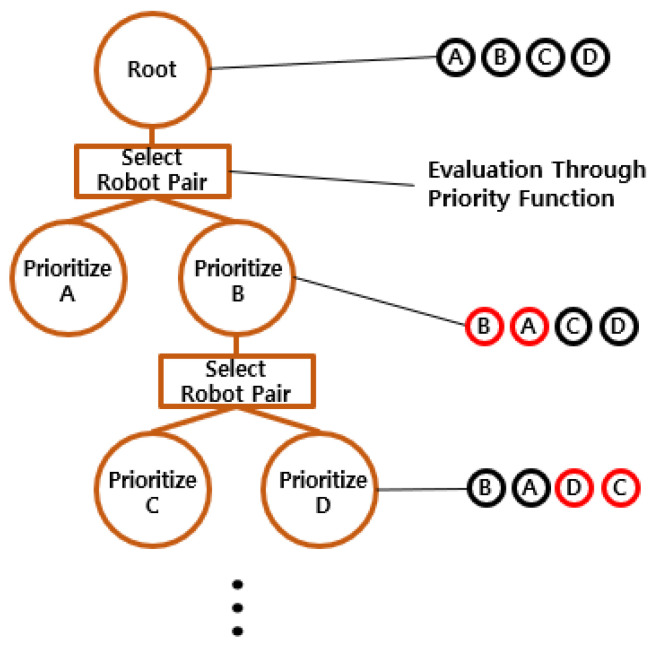
Example of priority tree search.

In the root node, the priorities of robots are initially assigned based on the request times of their allocated tasks. A priority evaluation function is then used to select a pair of robots for potential reordering. Figure 6 shows an example with the original task order given as ABCD. If the evaluation function selects the pair (A, B), priority order of the two robots is swapped, and new paths are computed through the low-level search. If the new plan brings improvement to the solution, the updated order is adopted. Otherwise, the original order is retained. The PT is branched following the result of the reordering. The evaluation function is then used again to select the next pair, such as (C, D), and the same process is repeated. The iterative refinement continues to explore improved solutions for multiple parking robots.

PBS is designed for handling a large number of robots, using depth-first search without evaluating intermediate solutions. The search terminates as soon as any valid solution is found. The approach can be effective in scenarios where real-time planning for thousands of agents is required. However, parking lot environments do not involve such a large number of robot fleets. Instead, the objective is to complete as many tasks as possible without making customers wait too long using a relatively small number of robots. Therefore, the proposed method departs from PBS by incorporating a solution evaluation process after each iteration. We also adopted a best-first search to improve the quality of the resulting plan.

### 3.2. Priority Evaluation Function

The priority evaluation function $f_{\mathrm{pair}}$ was designed to select a robot pair for priority reordering in the high-level search. The function assesses each robot pair by considering two factors. The first is the degree of fairness violation that would result from changing their order. The second is the potential efficiency gain, reflecting how reordering priorities can lead to more efficient task execution. Robot pairs are ranked based on the evaluation. The pair with the highest rank is then selected, and the PT is expanded by the result of order modification.

(1) $$f_{\mathrm{pair}}=w_{\mathrm{priority}}*C_{\mathrm{priority}}+w_{\mathrm{detour}}*C_{\mathrm{detour}}$$

The priority evaluation function consists of the following components.

- $C_{\mathrm{priority}}$ represents the priority cost incurred by reordering two robots. It quantifies the violation of task request orders to reflect fairness in customer waiting. A smaller time difference between task requests results in less significant fairness violations. Priority cost is computed as the time difference between a robot with an earlier task request and one with a later request, yielding a negative value.
- $C_{\mathrm{detour}}$ denotes the detour cost caused by additional path length due to collision avoidance when the priority remains unchanged. Detour cost captures efficiency degradation. When a detouring maneuver requires a significantly longer travel time for a lower-priority robot, changing the priority becomes more favorable. The detour cost is calculated as the difference in travel time between the detouring and non-detouring paths, resulting in a positive value.
- $w_{\mathrm{priority}}$ and $w_{\mathrm{detour}}$ are the weights assigned to $C_{\mathrm{priority}}$ and $C_{\mathrm{detour}}$, respectively, with the constraint $w_{\mathrm{priority}}+w_{\mathrm{detour}}=1$. Adjusting the weights allows the search process to reflect the relative importance of priority and efficiency.

By applying this evaluation function, the proposed path planner can generate solutions that balance operational efficiency with fairness in customer waiting.

### 3.3. Low-Level Search

**Figure 7:**
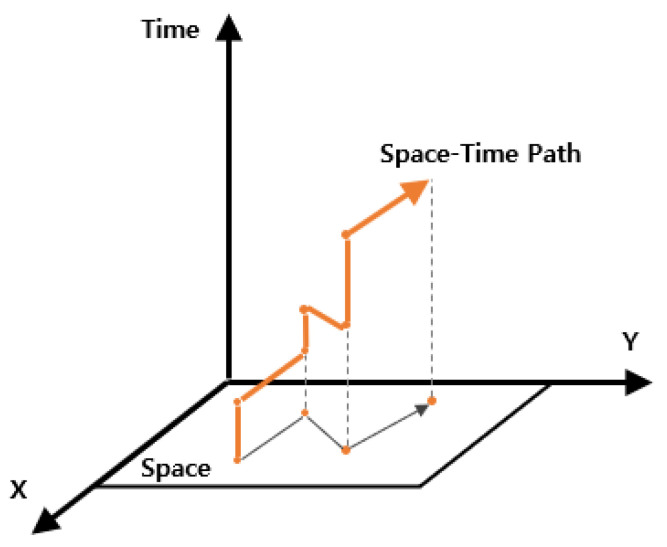
Space–time path from low-level search.

In the low-level search, a space–time path for a single robot consisting of x, y, and time is planned, as illustrated in Figure 7. Path planning follows the priority order determined at the high level, starting from a robot with the highest priority. Each planned path is stored in a reservation table. Robots with lower priority search for their paths while avoiding conflicts with higher-priority paths. No existing algorithm could directly support the characteristics of the aforementioned parking robot. Therefore, a custom single-robot path planning method was developed to reflect these dynamics in the low-level search.

A*-based path planning methods construct paths by storing parent nodes during the search and tracing them backward once the goal is reached [26]. However, since only adjacent nodes can be considered parent nodes, accounting for the time consumed by acceleration, deceleration, or rotation cannot be properly reflected. As a result, shortest-distance paths that frequently lead to longer execution times are often generated.

The developed single-robot path planning method extends the any-angle search approach from [27] by allowing each node to have multiple parent candidates. All nodes that can reach the current node via a straight path are considered parent candidates. Among the candidates, the node that enables the fastest arrival time is selected as the parent node. In the process, the arrival time is computed based on the actual motion of the robot. Therefore, the planner can generate paths reflecting realistic behaviors such as acceleration, deceleration, and rotation. As a result, low-level search can find a minimum time path that is not necessarily the shortest distance path. Furthermore, the planner searches over a continuous time window rather than discrete time steps to allow for precise temporal planning.

A new solution from the low-level search is accepted only if it improves the previous one. The evaluation metrics used to determine such improvements are discussed in Section 4. The search adheres to the principle that priority relations established in the upper nodes of the priority tree must remain fixed in their descendant nodes. Through the searching process, the proposed method enables path planning in high-density parking environments while balancing both priority and efficiency.

## 4. Evaluation

### 4.1. Experiment Setup

**Figure 8:**
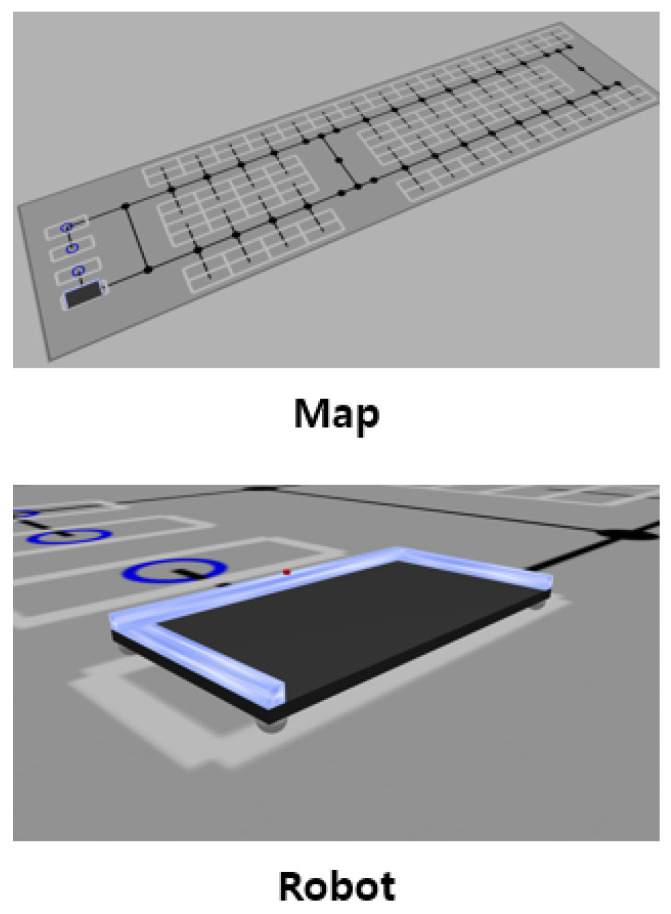
ROS 2 simulator environment.

**Figure 9:**
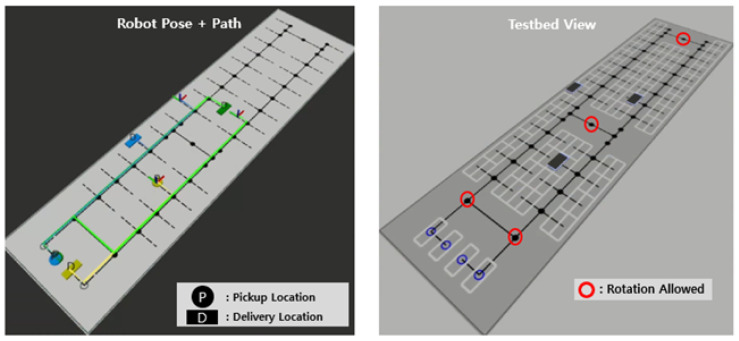
Experiment using the simulator.

A parking lot simulation environment was developed to evaluate the proposed path planning method. The simulator was built on ROS2 and Gazebo. The topological map shown in Figure 8 replicates the actual scale of the parking testbed built at Incheon Airport. The real-world parking robot currently under development lifts vehicles from the side using a gantry mechanism, similar to a forklift. Two separate gantries are equipped to lift both the front and rear wheels independently. The robot is capable of omnidirectional movement by adjusting the orientation of its wheel axes. In addition, the robot is equipped with cameras and LiDAR sensors to perceive the current position and recognize vehicle wheels accurately. The parking robot was simulated with URDF according to its real-world properties such as size and velocity. The omnidirectional mobility of the robot was also accurately incorporated into the simulation. Experiments were conducted as in Figure 9 using the developed simulator.

**Figure 10:**
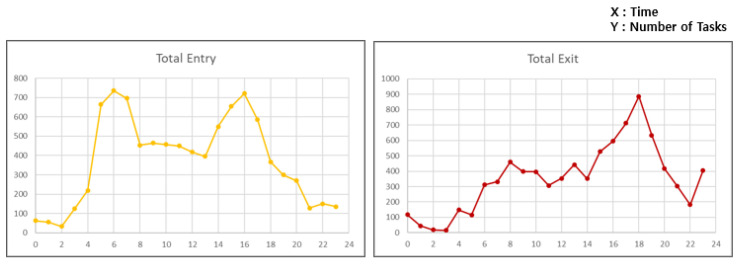
Peak season parking entry and exit data.

Real-world parking data from peak season was used as input to the simulator. As shown in Figure 10, the dataset includes all vehicle entries and exits over a 24 h period during the peak month of 2019, covering 3800 parking slots. Since the parking testbed includes only 100 parking spaces, the dataset was downsampled by a factor of 1/38 for proper simulation. The original data shows peak a hour task request rate of up to 1536 vehicles per hour. In order to resolve congestion in the parking lot, the system with 100 parking slots must support a throughput of over 41 vehicles per hour during peak demand.

### 4.2. Evaluation Metric

In order to evaluate the proposed method, two metrics were selected, representing priority and efficiency, respectively. First, throughput was used as an indicator of task efficiency. Throughput is defined as the number of completed parking operations per unit time. Higher throughput indicates greater operational efficiency. The metric is commonly adopted in MAPF literature as well as in studies on parking robot systems.

Next, fairness was defined as a measure of consistency between task execution order and the customer request order. Fairness is calculated as the ratio of tasks completed in the same order as their original request order. In other words, it measures the proportion of tasks that were processed in the requested temporal order. A higher fairness value indicates a greater level of equity in handling customer requests.

### 4.3. Experiment Result

**Figure 11:**
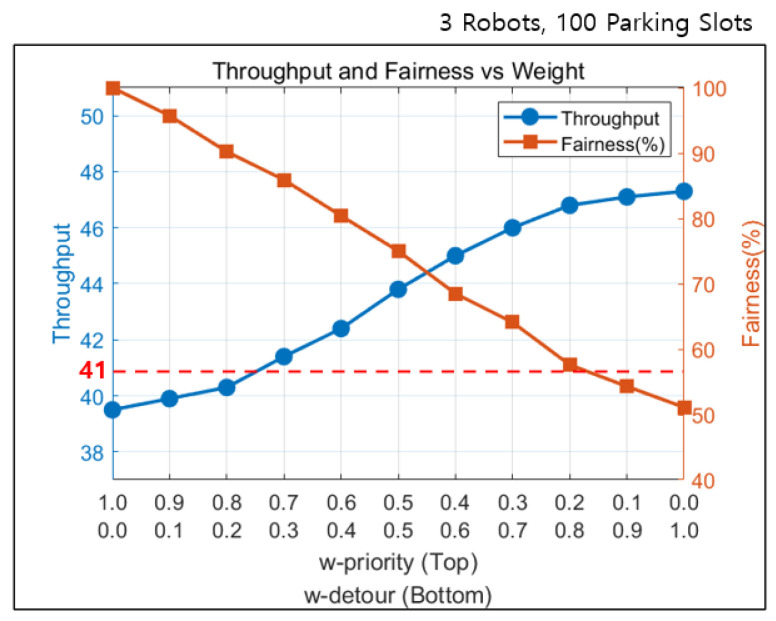
Experimental results of the proposed method.

The experiment result of the proposed method evaluated in the developed simulation environment is shown in Figure 11. The blue line represents throughput, and the orange line represents fairness. The y-axis on the left side corresponds to throughput, and the y-axis on the right side indicates fairness as a percentage. When $w_{\mathrm{priority}}$ is high, the system prioritizes fairness, resulting in lower throughput. Such setting does not allow the throughput to meet the peak season demand of 41 vehicles per hour. In contrast, setting $w_{\mathrm{detour}}$ higher improves efficiency but leads to increased violations of requested task order. The proposed method achieves a throughput that exceeds 41 vehicles per hour when $w_{\mathrm{priority}}$ is set below 0.7. With such settings, we have confirmed that the system can handle peak season demand through appropriate weight adjustment.

The input data shows a significant variation in demand throughout the day. The number of tasks increased dramatically during peak hours. On the other hand, the workload was less than one-tenth of that in the least busy hours, allowing for more flexibility in operation. By applying the proposed method to airport operations, it is possible to establish parking strategies depending on the time of day. In the least busy hours, the system can prioritize task orders by assigning weights that favor fairness. During peak hours, the weights can be adjusted to increase throughput and improve overall processing efficiency. Accordingly, the proposed method can be deployed to enable adaptive operation based on time-dependent demand patterns.

**Figure 12:**
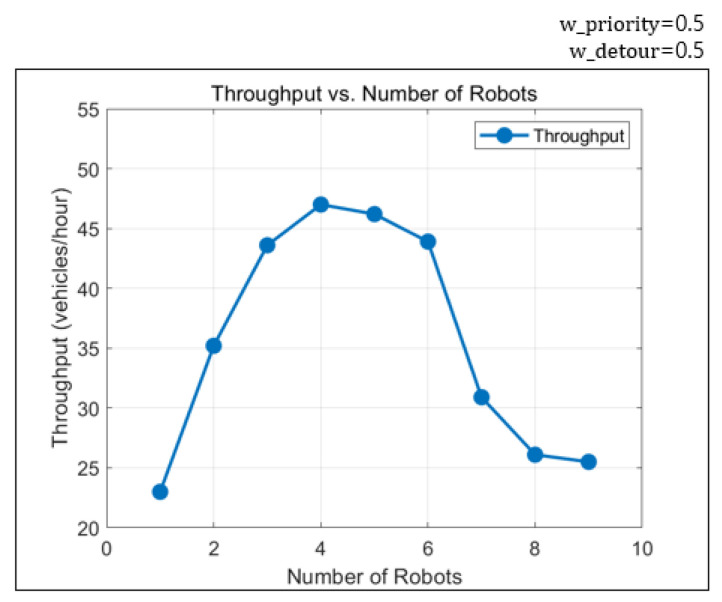
Throughput variation with respect to the number of robots.

The HDP environment for parking robots has narrow corridors and designated entry and exit stations. Hence, increasing the number of robots does not always lead to improvement in efficiency. When unnecessarily large numbers of robots are deployed, the robots may block paths of each other, resulting in failure to find valid solutions. Moreover, while the operational cost grows proportionally with the number of robots, the throughput does not always scale accordingly. Therefore, identifying the appropriate number of robots for a given parking environment beforehand is essential. In the parking lot testbed with 100 slots used in the simulator, the highest throughput was achieved with four robots. Figure 12 shows the experimental result. The planner frequently failed to find a solution when the number of robots exceeded seven. Throughput decreased due to congestion even in successful cases. Using the proposed method and simulator, it is possible to estimate the recommended number of robots for a given parking environment.

## 5. Conclusions

The proposed method addresses multi-robot path planning in high-density parking environments by considering both efficiency and individual task priority. The method was validated using a simulation environment modeled after a real-world autonomous parking testbed. In this study, we extended the MAPF framework from the original warehouse applications to parking scenarios. The proposed method can generate paths balancing operational efficiency with customer-oriented task priority. A single robot planner was developed to reflect the dynamic behavior of real parking robots, allowing for accurate search based on actual travel times. Through experiments based on peak season parking data from Incheon Airport, we showed that high parking demand can be handled by adjusting weight values. In addition, performance evaluations can estimate the recommended number of robots and achievable throughput across different parking environments.

While existing MAPF methods such as PBS and TP-SIPPwRT offer effective priority-based planning, they are not applicable to our scenario. PBS assumes ideal agent behavior, and the motion dynamics of real robots are not considered. This limitation is critical in our scenario, where the parking robots take some time to accelerate and need to stop before rotation. As a result, infeasible or unsafe trajectories were generated under our robot model, making direct performance comparison impractical. TP-SIPPwRT also could not account for these characteristics, resulting in a similar situation. Moreover, the decentralized design of TP-SIPPwRT is optimized for large-scale systems without centralized control. The framework in our study operates within a structured environment with reliable communication between the server and robots.

An automated parking site is currently under construction at Incheon Airport for experiments in a real-world environment. In order to extend the simulation results to accommodate uncertainties in actual environments, several considerations can be made. First, robots may arrive at the goal node later than expected, potentially causing collisions between robots. The low-level search can be modified to include extended occupancy time intervals as a buffer to address the delay. In cases of significant delays, re-planning might become necessary. Next, for further applications, the system can also account for unforeseen obstacles not included in the map. In such cases, robots can trigger an emergency stop based on the sensor input, close the affected edge, and initiate a re-planning process. Furthermore, to mitigate sensor errors in real-world environments, the physical layout can be adjusted by slightly increasing the spacing between driveways and parking slots.

Scenarios involving vehicle blockage due to multiple parking rows were not considered in the current work but need to be addressed for the practical deployment of HDP systems. We also assumed that tasks and parking slots were assigned beforehand using simplified modules. Enhancing both the task and slot assignment processes could further improve the efficiency of parking entry and exit tasks. The proposed approach has the potential for broader application to resolve parking issues in congested urban areas such as offices or restaurants.

## Data Availability

Data are contained within the article.

## Abbreviations

The following abbreviations are used in this manuscript:

HDP	High-Density Parking
MAPF	Multi-Agent Path Finding
PBS	Priority-Based Search
CBS	Conflict-Based Search
SIPP	Safe Interval Path Planning
CA*	Cooperative A*
PT	Priority Tree
